# Influence of Sampling Rate on Wearable IMU Orientation Estimation Accuracy for Human Movement Analysis

**DOI:** 10.3390/s25071976

**Published:** 2025-03-22

**Authors:** Bingfei Fan, Luobin Zhang, Shibo Cai, Mingyu Du, Tao Liu, Qingguo Li, Peter Shull

**Affiliations:** 1 College of Mechanical Engineering, Zhejiang University of Technology, Hangzhou 310014, China; bingfeifan@zjut.edu.cn (B.F.);; 2 Key Laboratory of Special Purpose Equipment and Advanced Processing Technology, Ministry of Education and Zhejiang Province, College of Mechanical Engineering, Zhejiang University of Technology, Hangzhou 310014, China; 3 State Key Laboratory of Fluid Power and Mechatronic Systems, School of Mechanical Engineering, Zhejiang University, Hangzhou 310027, China; 4 Department of Mechanical and Materials Engineering, Queen’s University, Kingston, ON K7L 3N6, Canada; 5 State Key Laboratory of Mechanical System and Vibration, School of Mechanical Engineering, Shanghai Jiao Tong University, Shanghai 200240, China

**Keywords:** inertial measurement unit, strap-down integration, high-rate gyroscope, orientation estimation, sensor fusion algorithms, sampling rate influence

## Abstract

Wearable inertial measurement units (IMUs) have been widely used in human movement analysis outside the laboratory. However, the IMU-based orientation estimation remains challenging, particularly in scenarios involving relatively fast movements. Increased sampling rate has the potential to improve accuracy, but it also increases power consumption and computational complexity. The relationship between sampling frequencies and accuracies remains unclear. We thus investigated the specific influence of IMU sampling frequency on orientation estimation across a spectrum of movement speeds and recommended sufficient sampling rates. Seventeen healthy subjects wore IMUs on their thigh, shank, and foot and performed walking (1.2 m/s) and running (2.2 m/s) trials on a treadmill, and a motion testbed with an IMU was used to mimic high-frequency cyclic human movements up to 3.0 Hz. Four widely used IMU sensor fusion algorithms computed orientations at 10, 25, 50, 100, 200, 400, 800, and 1600 Hz and were compared with marker-based optical motion capture (OMC) orientations to determine accuracy. Results suggest that the sufficient IMU sampling rate for walking is 100 Hz, running is 200 Hz, and high-speed cyclic movements is 400 Hz. The accelerometer sampling rate is less important than the gyroscope sampling rate. Further, accelerometer sampling rates exceeding 100 Hz even resulted in decreased accuracy because excessive orientation updates using distorted accelerations and angular velocity introduced more error than merely using angular velocity. These findings could serve as a foundation to inform wearable IMU development or selection across a spectrum of human gait movement speeds.

## 1. Introduction

Wearable inertial measurement units (IMUs) are promising tools in ambulatory human movement analysis [1,2] for their advantages such as their small size, low cost, and light weight. IMUs have been widely used in gait analysis, rehabilitation training, ergonomics, and sports [3,4]. For example, IMUs have been employed in calculating spatiotemporal gait parameters, knee kinematics, and full-body joint angles to diagnose gait disorders [5], assess the risk of anterior cruciate ligament injury [6,7], and capture body movements for game control [8]. In most of these applications, accurate IMU orientation estimation is critical. Some applications used the orientation directly for segment orientation or joint angle measurement [9,10], while others used orientation to derive linear acceleration by removing the gravity component from the measured acceleration and, subsequently, calculating the displacement through double integration of the linear acceleration [11,12]. Errors in orientation estimation may lead to significant error accumulation in subsequent estimations of joint angles or displacements [13]. Therefore, accurate orientation estimation is extremely important for IMU-based human movement analysis.

Despite numerous sensor fusion algorithms (SFAs) proposed to estimate IMU orientation by fusing the measured acceleration, angular velocity, and magnetic field [14], achieving accurate orientation estimation in human motion analysis remains challenging. There are two common strategies to improve the accuracy of orientation estimation. The first class of methods focuses on sensor calibration, usually using an instrumented turntable to calibrate raw sensor measurements, thereby enhancing the accuracy of acceleration, angular velocity, and magnetic field measurements [15]. Another alternative is to improve the SFAs to fully exploit the complementary characteristics of angular velocity integration-based and accelerometer/magnetometer-based orientation calculations [16]. Many studies have developed different SFAs to enhance orientation estimations. For example, Madgwick et al. [17] introduced the gradient descent algorithm and subsequently developed an extended complementary filter [18], both of which have been extensively adopted in the literature. Nazarahari et al. [19] implemented 36 SFAs and compared their performance under various conditions. Recently, Laidig et al. [20] proposed a versatile quaternion-based inertial orientation estimation filter (VQF) that incorporates features of gyro-bias estimation and magnetic disturbance rejection. However, the accuracy of the orientation estimation reaches a limit. Even in the results of the latest studies, orientation errors could frequently exceed 5°, particularly during dynamic movement and under magnetic disturbance [19]. Achieving accurate orientation estimation in human motion analysis remains a significant challenge.

Gyroscopes are crucial for accurate orientation estimation. For SFAs, the ideal situation of orientation estimation is that accelerometers only measure gravity acceleration, and magnetometers only measure the geomagnetic field. However, in practical applications, accelerometers measure not only gravity acceleration but also dynamic movement acceleration. Achieving homogeneous geomagnetic field measurements necessitates not only a magnetically clean environment but also careful magnetometer calibration. Any nearby ferromagnetic materials in our surroundings [21], improper magnetometer calibration, or prolonged exposure to a strong magnetic field environment [22] can lead to non-homogeneous magnetometer measurements, causing yaw errors in orientation estimation. When accelerometer and magnetometer measurements become unreliable, angular velocity integration becomes the sole means to achieve accurate orientation estimation, highlighting the importance of gyroscopes in orientation estimation. Furthermore, most off-the-shelf IMUs typically have data output rates ranging from 100 to 200 Hz, and the SFAs presented in the literature were often tested under these frequencies [23,24,25]. Although increasing the gyroscope data rates is a potential strategy to enhance orientation estimation accuracy, the influence of IMU sampling frequency on orientation estimation in human motion analysis remains unclear.

In addition, most wearable IMU systems are powered by a battery. Thus, power consumption is a crucial determinant of the battery life, affecting the system’s usability. The power consumption can be reduced using lower IMU sampling frequency and less complex algorithms [26]. Williamson et al. [27] reported that the average power consumption positively correlated with sampling frequencies. Carrat et al. [28] found that the complex Kalman filter consumes about 29% more energy than the simpler quaternion filter. Lower IMU sampling frequency can also decrease the wireless data transfer volume, thus enhancing the reliability of an IMU system [29]. Therefore, it is important to explore acceptable lower sampling frequencies for specific human movements to obtain a good compromise between accuracy and sampling frequency and to reduce the computational complexity of a sensor fusion algorithm.

This study aims to investigate the influence of IMU sampling frequency on orientation estimation accuracy across various SFAs and to suggest appropriate sampling frequencies for various human movement analyses. We collected high-frequency IMU data on the thigh, shank, and foot during walking, running, and on a cyclic movement testbed. Subsequently, we evaluated the orientation estimation accuracy of state-of-the-art SFAs by comparing the results with the optical motion capture (OMC). The main contribution of this work is two-fold. First, we provided insight into how IMU sampling rate affects the orientation estimation and recommended a sufficient IMU sampling rate for different movement speeds. Second, we recommended an improved strap-down integration approach that utilizes high-frequency gyroscope data for low-frequency SFAs while maintaining accuracy.

## 2. Materials and Methods

### 2.1. Measurement Systems

We used commercially available high-frequency MTi-630 IMUs (XSENS Technologies B.V., Enschede, The Netherlands) for data collection, which contain a 3-axis gyroscope (range ± 2000 deg/s), a 3-axis accelerometer (range ± 10 g), and a 3-axis magnetometer (range ± 8 Gauss). This IMU supports high output data rate modes. The maximum data rate of the gyroscope is 1600 Hz, the acceleration is 2000 Hz, and the magnetometer is 100 Hz. These IMUs were connected to a PC via USB cables, and the baud rate was set to 2 Mbit/s. The sampling rate of the gyroscope was set to the maximum frequency of 1600 Hz, and the accelerometer was set to 1000 Hz to avoid possible data overflow during transmission. The acceleration data were spline-interpolated to 1600 Hz to match the gyroscope data rate. Both of the datasets were subsequently downsampled to 800 Hz, 400 Hz, 200 Hz, 100 Hz, 50 Hz, 25 Hz, and 10 Hz by picking data points from the original dataset to construct a new dataset for analyzing the influence of sampling frequency in sensor fusion. Two orientation estimation experiments were performed under separate conditions.

#### 2.1.1. Experimental Setup for Human Subjects Test

**Figure 1:**
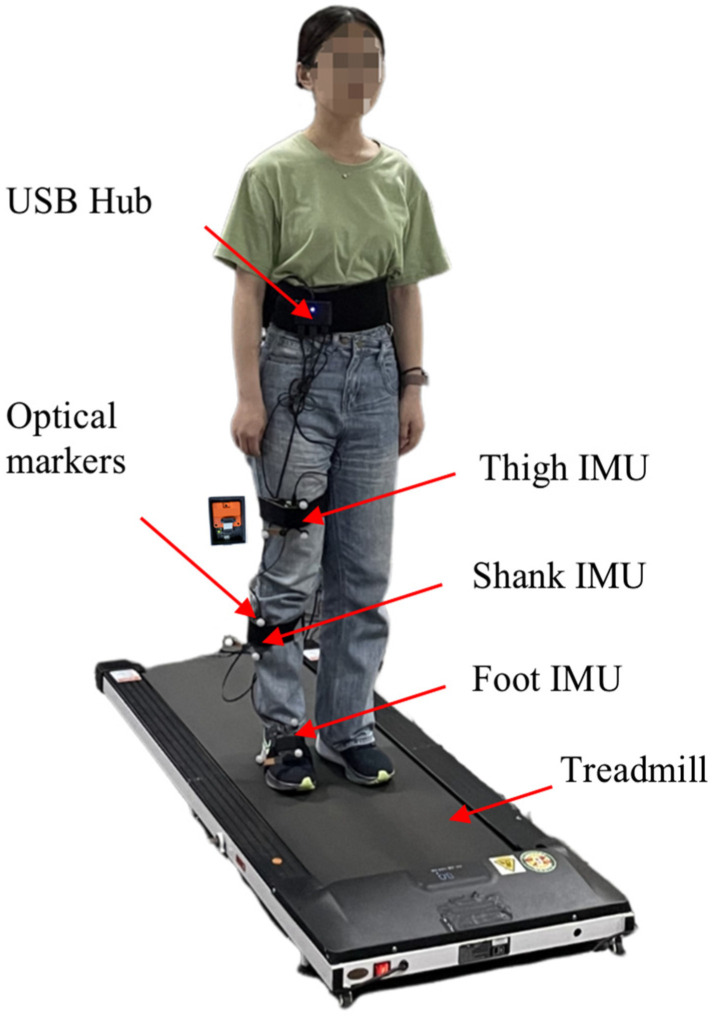
Experimental setup for walking and running trials. Three IMUs were attached to the thigh, shank, and foot. Each IMU was fixed with a marker cluster comprising three markers for reference orientation calculation.

The first experiment was orientation estimation during human walking and running. Three IMUs were attached to the subject’s thigh, shank, and foot (Figure 1). All IMUs were connected to a USB hub, which was then connected to the computer. An optical motion capture system with 12 Mars 2H cameras was used as the reference system (NOKOV Motion Capture, Beijing, China) [30], with a sampling rate of 100 Hz. The captured marker position accuracy was ±0.15 mm. Each IMU was fixed with a marker cluster comprising three markers. The orientation of the marker cluster was used as the gold standard, which was computed using Schmidt orthogonalization based on the position data of the markers [31]. We estimated that the marker position errors caused a maximal error of 0.3° using the geometry method. This error is often regarded as acceptable for reference angle [32]. The initial offset between IMU orientation and marker cluster orientation was calculated and compensated based on their initial orientations. During the OMC system’s calibration process, the *X*-axis of OMC was aligned with the north to maintain consistency with the IMU frame. The IMU and OMC systems were manually synchronized by identifying the peaks of their respective Euler angles.

#### 2.1.2. Experimental Setup for Cyclic Movements Test

**Figure 2:**
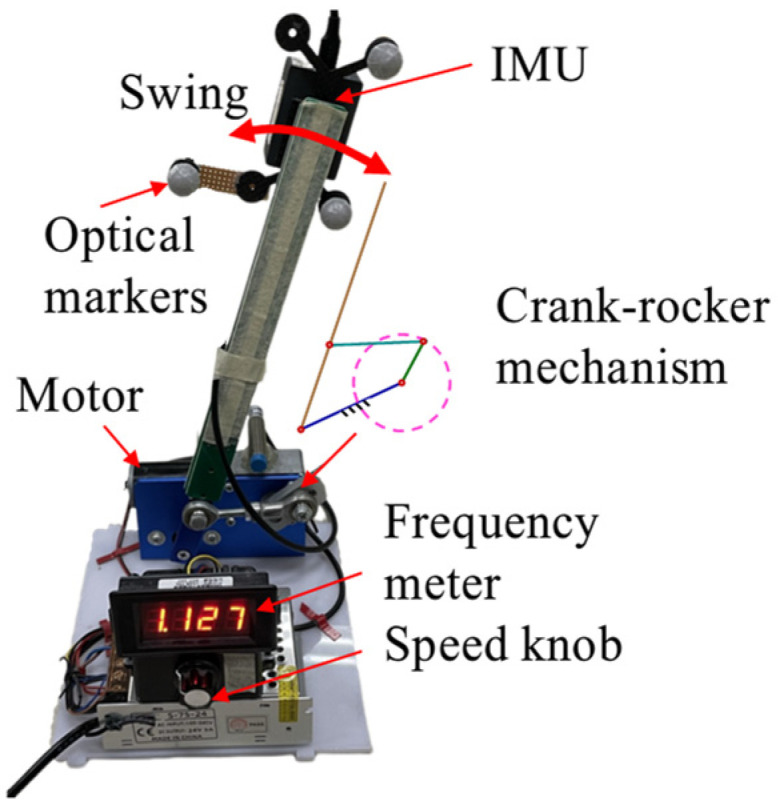
Experimental setup for the motion testbed. A customized motion testbed was used to mimic cyclic human movements, with movement frequencies set at 1 Hz and 3 Hz.

The second experiment was orientation estimation under cyclic movements. A customized motion testbed (Figure 2) was used to mimic cyclic human movements such as walking and running. We added this experiment because testing an SFA under a severe condition was necessary, and this device provided a repeatable, safe, severe condition. The testbed was driven by a DC motor. A frequency meter was used to measure the swing frequency of the arm, and a speed knob was used to regulate the swing frequency between 0 and 5 Hz. Because the maximum stride frequency was about 2.2 Hz [33], we set the maximum test frequency to 3 Hz and a normal motion frequency to 1 Hz. Before testing, the IMU and the optical marker cluster were fixed to the end of the arm. This customized testbed could be easily controlled and set to desired frequencies, enabling the imitation of various cyclic movements.

### 2.2. Experimental Protocol

#### 2.2.1. Orientation Estimation on Human Subjects

Seventeen healthy male/female subjects participated in this experiment (12 male, 5 female, age: 25.8 ± 4.1 years, height: 172.2 ± 7.5 cm, weight: 66.0 ± 10.7 kg). None of the subjects had a history of lower limb injury or systemic neuromuscular disease. Each subject completed two trials: walking and running on a treadmill. Each trial consisted of standing still for 10 s, followed by walking or running for 120 s, and finally resting for 10 s. The walking speed was set at 1.2 m/s, and the running speed was set at 2.2 m/s. The experiment adhered to the Helsinki Declaration and received approval from the institutional review board (KY2022-173). Informed written consent was obtained from all subjects before data collection.

#### 2.2.2. Orientation Estimation on the Motion Testbed

In this experiment, the IMU was kept stationary for 10 s, followed by shaking the motion testbed at 1 Hz for 30 s. After another 10-s stationary period, the motion testbed was shaken at 3 Hz for 60 s, thus mimicking cyclic movements at moderate frequencies [34]. This trial was repeated ten times.

### 2.3. Sensor Fusion Algorithms

IMU SFAs continually evolve in the field of human movement analysis. Recently, several SFAs have been proposed with reported higher accuracy [14,20,23]. For this study, we selected four representative SFAs: the FSM algorithm [11], Madgwick’s improved extended complementary filter [18], VQF [20,35], and the SEL [36]. These algorithms are popular and easily available. The details of these selected algorithms are described as follows:

The FSM algorithm [11] is a two-step complementary filter that decouples attitude and heading estimation. The first step updates orientation using gyroscope and acceleration data, while the second step corrects only the heading yaw angle, thereby decoupling the effect of magnetic disturbance on attitude angle.

Madgwick et al. [18] introduced a new algorithm in 2020, an extended complementary filter (ECF), which combines the computational efficiency of classic complementary filters with improved accuracy compared to the previous gradient descent algorithm. This algorithm introduced a special step in calculating the error between measured east and predicted east, avoiding magnetic interference on pitch and roll estimation. ECF has been validated in a commercial product released by x-io Technologies.

The VQF (versatile quaternion-based filter) is a novel approach that filters acceleration measurements in an inertial frame [20,35]. The basic functions of the VQF include strap-down integration, inclination correction, and heading correction. Additional functions include rest state detection, gyroscope bias estimation, and magnetic disturbance rejection. The VQF had been validated on several open datasets, and the results outperformed those of eight selected methods [20]. The VQF has accompanied open-source implementations that facilitate its use in human motion analysis.

Thomas Seel et al. [23,36] proposed an SFA that eliminates the effect of magnetic disturbances on attitude estimates. The SEL (SEL) incorporates a gyro-based prediction step and two correction steps: an accelerometer-based correction step and a magnetometer-based correction step, which calculates the correction quaternion and corrects the error of gyro-based prediction. Each correction step contains a time constant to regulate correction speed. The magnetometer-based correction step only corrects the yaw angle, thus affecting only yaw angle estimation.

Additionally, angular velocity integration (INT) is critical in SFAs, especially when accelerometer and magnetometer readings are unreliable. Although INT cannot be used as an independent SFA because it requires an initial orientation and is accurate only over a short period (several seconds), we used INT as a comparison algorithm with an initial angle calculated from acceleration and angular velocity during the stationary state. We also investigated the influence of different integration methods on orientation estimation. These integration methods can be categorized into two types:

1. Quaternion addition-based integration method (${INT}_{add}$) [17]

The ${INT}_{add}$ method is described in (1) to (4). The key step of quaternion iteration is quaternion addition, (3). This method is widely used in SFAs [11,17,37].

(1) $$S_{\omega_{t}}=[\begin{array}{cccc} 0 & \omega_{x} & \omega_{y} & \omega_{z} \end{array}],$$

(2) q˙mES=12q^m−1ES⨂Sωt,

(3) qm′ES=q^m−1ES+q˙mESΔt,

(4) qmES=qm′ES/Norm(qm′ES).

2. Quaternion multiply based integration method (${INT}_{multiply}$)

The ${INT}_{multiply}$ method converts angular velocity to a quaternion increment [38], ∆q, which is defined as follows:

(5) $$∆q=\left(\begin{array}{c} cos\left(0.5\phi_{m}\right)\\ \frac{sin\left(0.5\phi_{m}\right)}{0.5\phi_{m}}\cdot 0.5{\vec{\Phi }}_{m} \end{array}\right).$$

where $\phi_{m}$ is the magnitude of the rotation vector ${\;\vec{\Phi }}_{m}$. ${\vec{\Phi }}_{m}$ is defined as follows:

(6) $${\vec{\Phi }}_{m}=\int_{t_{m-1}}^{t_{m}}\vec{\omega }dt.$$

Then, multiply the quaternion of the previous step to obtain the quaternion in the current step, as described by

(7) qmES=qm−1ES⨂∆q.

The final step of ${INT}_{multiply}$ is quaternion multiplication, ensuring that the iterated quaternion remains a unit quaternion. ${INT}_{multiply}$ has the advantage of merging multiple ∆q into a single ∆q. For example, suppose that the gyroscope data rate is 1600 Hz; the 1600 Hz gyroscope data can be converted into 1600 Hz ${∆q}_{1600Hz}$, then fed into the SFAs for high-rate sensor fusion. Another approach is to merge four continuous high-rate $∆q{HR}_{1600Hz}$ into a ${∆q}_{400Hz}$, as described in (8), then feed them into SFAs for low-rate sensor fusion. Since the $∆q{LR}_{400Hz}$ and the multiplication of four ${∆q}_{1600Hz}$ are equivalent, the low-rate SFAs can achieve comparable accuracy to high-rate SFAs while requiring less computational load as low-rate SFAs do not necessitate high-rate corrections based on accelerometer and magnetometer data.

(8) $${∆q}_{400Hz}={∆q}_{1600Hz\_1}⨂{∆q}_{1600Hz\_2}⨂{∆q}_{1600Hz\_3}⨂{∆q}_{1600Hz\_4}.$$

Each selected algorithm has several tuning parameters. We set the parameters according to the recommendation of the original literature (Table 1).

Additionally, in the orientation estimation procedure, the pre-processing of gyroscope data was also important. We remove gyroscope bias using the no-motion estimation method [16,40] based on the assumption that the gyro bias was nearly constant over a short period.

### 2.4. Data Analysis

The orientation calculated from the optical motion capture system was considered the gold standard. Root Mean Square Errors (RMSEs) of the estimated orientation of different SFAs under various conditions were calculated for comparison, including walking, running, slow movements, and fast movements conditions. Orientation estimations of the selected SFAs during representative trials were demonstrated. Furthermore, we presented the results of the orientation estimation over short durations and at different sampling frequencies of the accelerometer to analyze the specific influences of the gyroscope and accelerometer sampling frequencies.

## 3. Results

### 3.1. Walking and Running Experiments

**Figure 3:**
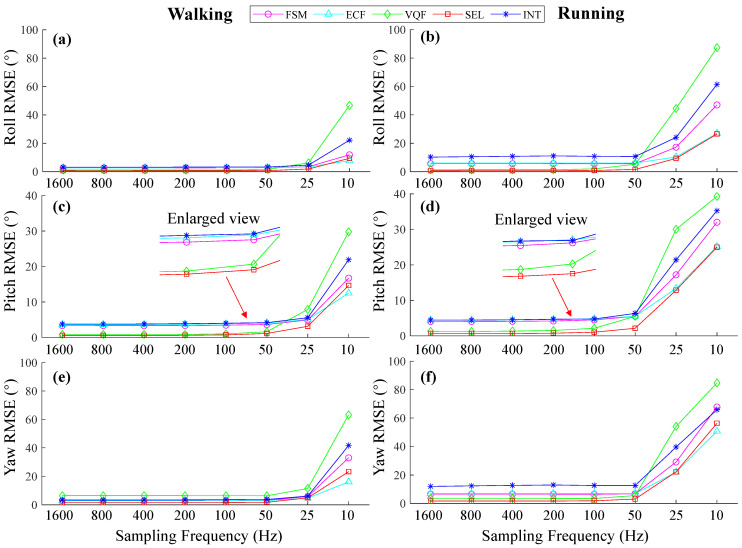
The orientation estimation of the selected SFAs with thigh, shank, and foot IMU data at different sampling frequencies. (**a**,**c**,**e**) Roll, pitch, and Yaw RMSEs of different SFAs under walking conditions. (**b**,**d**,**f**) Roll, pitch, and Yaw RMSEs of different SFAs under running conditions. The movement trials included continuous walking and running on the treadmill at speeds of 1.2 m/s and 2.2 m/s, respectively.

**Figure 4:**
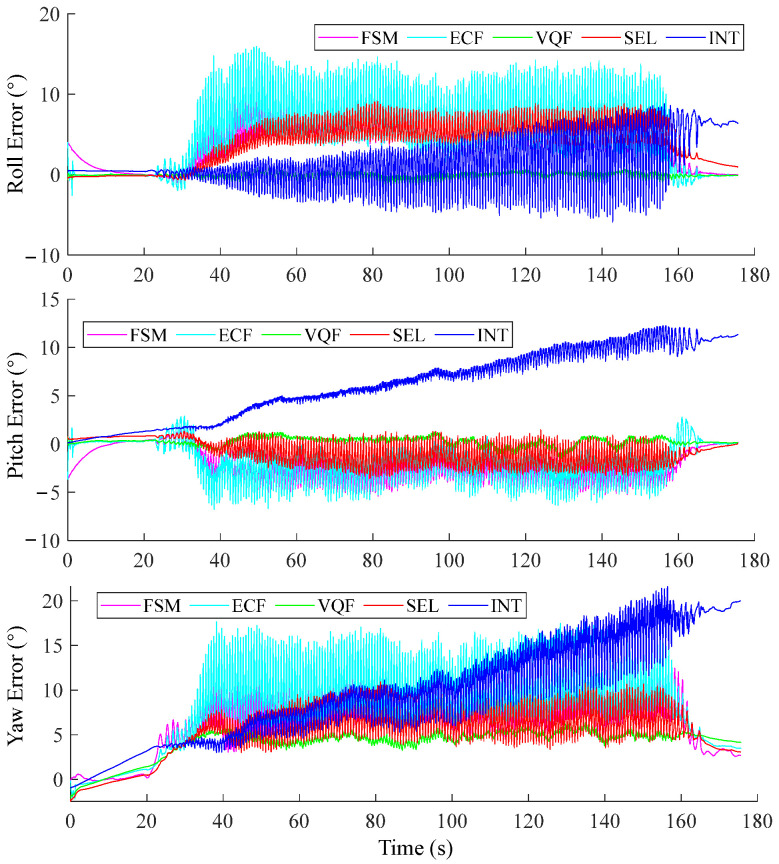
Foot IMU orientation estimation of the selected SFAs at 400 Hz sensor fusion. The movement trial was a 2.2 m/s running trial.

According to the results of the orientation estimation of the selected SFAs at various sampling frequencies during continuous walking and running trials (Figure 3), we can see that for walking, using frequencies above 100 Hz led to comparable accuracy, and the RMSEs increased when the frequencies fell below 100 Hz. For running, RMSEs were larger than those under walking conditions, and they increased when sampling frequencies fell below 200 Hz. In a representative trial during the running experiment, the VQF outperformed other SFAs (Figure 4).

### 3.2. Motion Testbed Experiments

**Figure 5:**
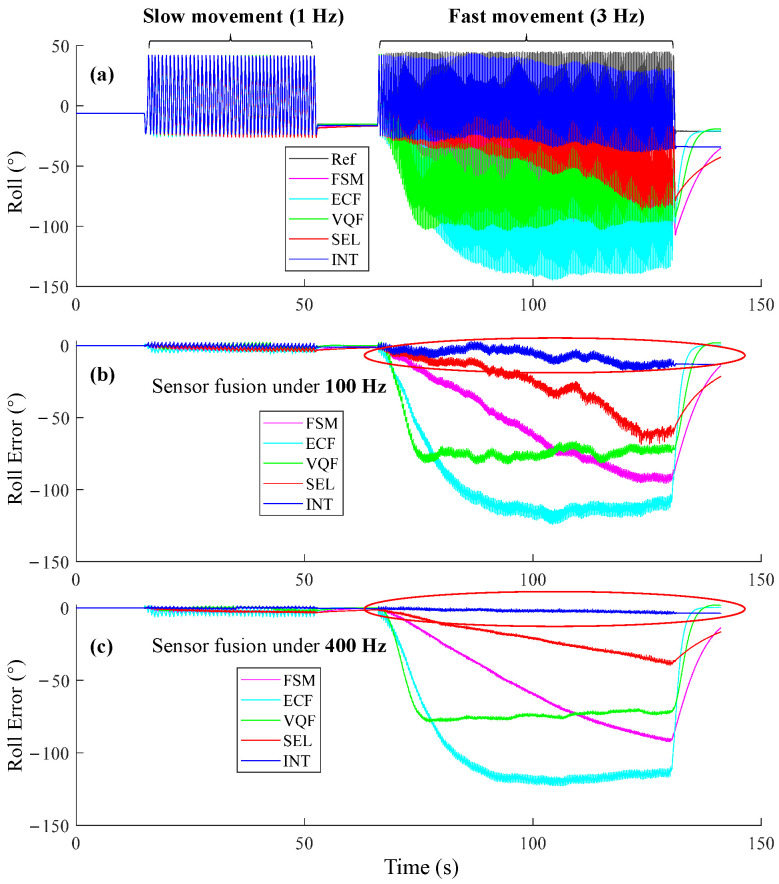
Orientation estimation of the selected representative SFAs. (**a**) Roll estimations at 100 Hz sensor fusion. (**b**) Roll error at 100 Hz. (**c**) Roll error at 400 Hz. The red circles highlight the estimation errors of INT under 100 Hz and 400 Hz.

During slow movements (1 Hz), all SFAs at 100 Hz exhibited comparable performance (Figure 5b). However, as movement frequencies increased, the estimated roll angle diverged. The errors of FSM, ECF, VQF, and SEL estimations reached up to dozens of degrees, which were unacceptable errors in human movement applications. Increasing the sensor fusion frequency to 400 Hz did not significantly reduce the errors but resulted in smoother error curves (Figure 5c). Interestingly, the presented INT method still performed well even in such challenging conditions, and the error at 400 Hz was smaller than that at 100 Hz.

**Figure 6:**
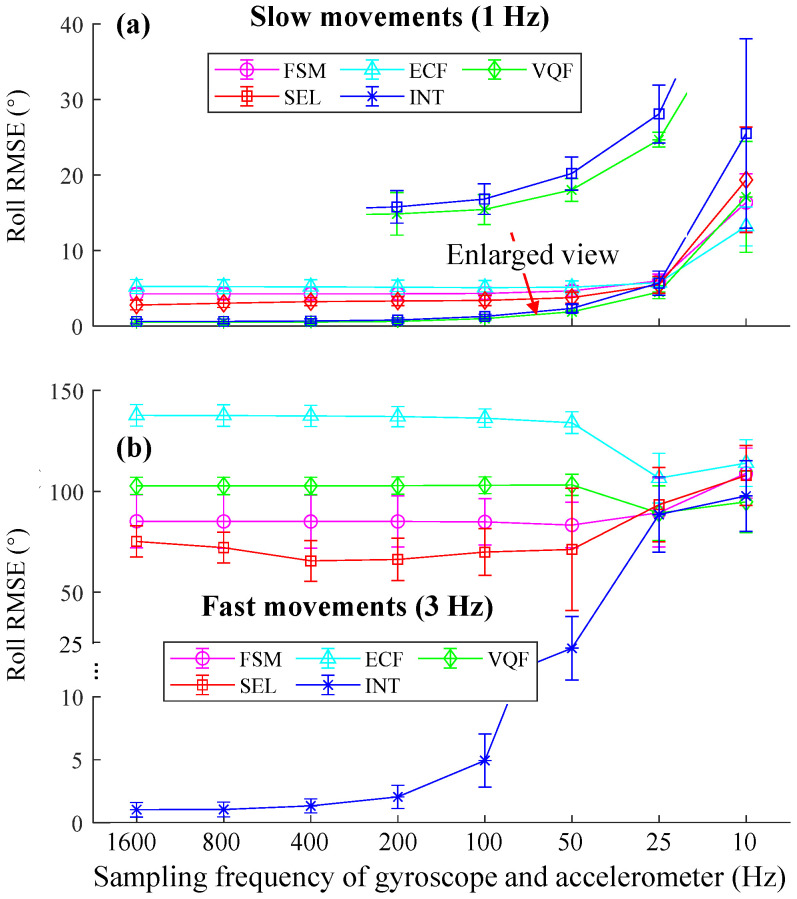
The mean and standard deviation of the roll RMSEs of different SFAs at different sampling frequencies. (**a**) Roll RMSEs under slow movement conditions. (**b**) Roll RMSEs under fast movement conditions.

The mean RMSEs of the roll estimations of different sensor fusion methods at slow movements (Figure 6a) indicate that using frequencies above 100 Hz led to comparable accuracy. Errors increased as frequencies decreased from 100 Hz to 10 Hz. While at fast movements, the errors of FSM, ECF, VQF, and SEL became tremendous (Figure 6b). The error of INT increased when the frequencies were smaller than 400 Hz.

**Figure 7:**
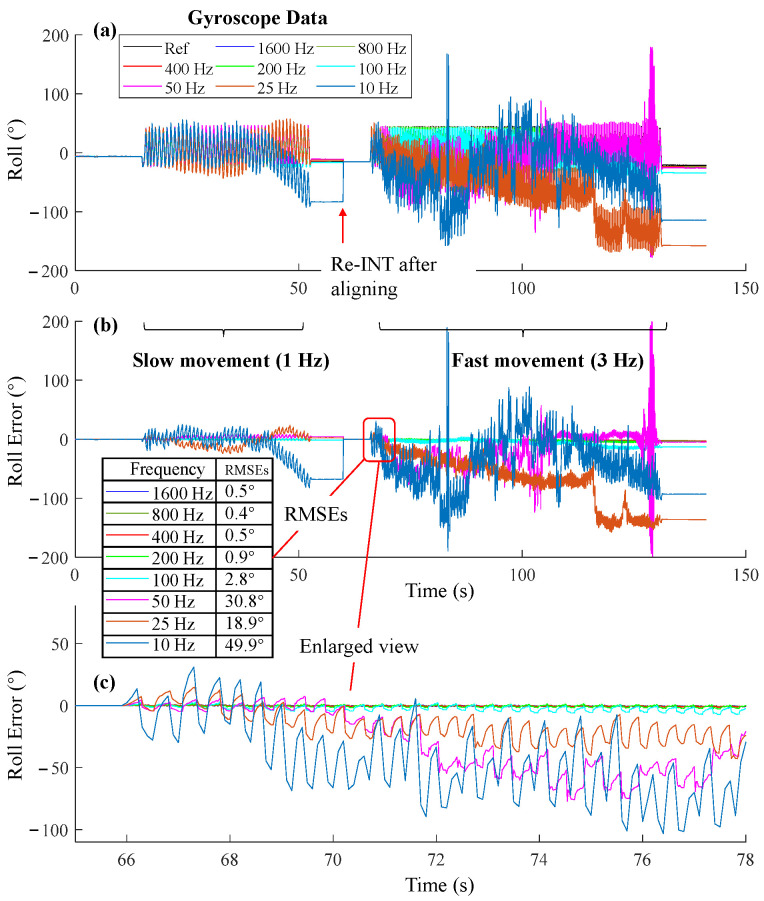
Orientation estimation of the INT algorithm at different sampling frequencies. The movement frequencies included 1 Hz and 3 Hz. (**a**) The estimated roll angles. (**b**) Roll error at different sampling frequencies. (**c**) Enlarged view at the initial period of fast movement.

We also presented the detailed results of the INT algorithm using angular velocities at different sampling frequencies (Figure 7). The RMSEs were calculated in the initial period to demonstrate the error trends. RMSEs tended to increase as the frequency decreased from 400 Hz to 10 Hz. RMSEs at 1600 Hz, 800 Hz, and 400 Hz exhibited comparable accuracy.

**Figure 8:**
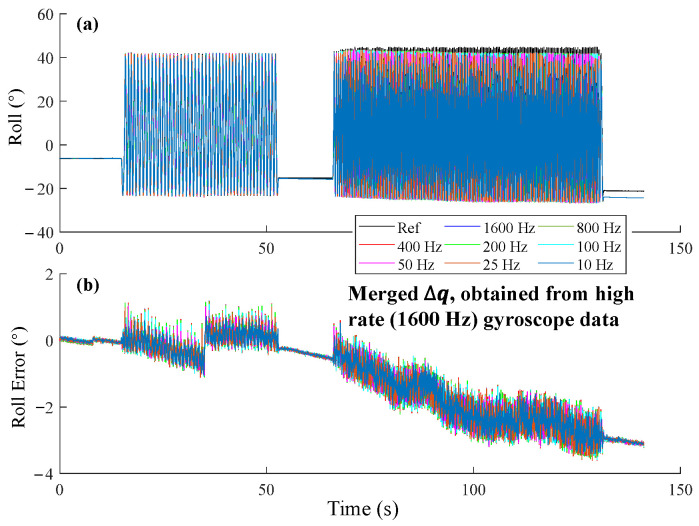
Orientation estimation of INT_merge_ at different integration frequencies, with the input ∆q merged from 1600 Hz gyroscope data. (**a**) The estimated roll angles. (**b**) Roll error at different integration frequencies.

Similarly, we used the INT algorithm but replaced the input data with a lower rate ∆q that was merged by multiplying several high-rate ${∆q}_{1600Hz}$. For example, ${∆q}_{400Hz}$ was obtained by the multiplication of four consecutive ${∆q}_{1600Hz}$, as described in (10). Here, we named this algorithm as INT_merge_. We observed that even at 10 Hz, INT_merge_ still achieved roll estimations similar to those at 1600 Hz (Figure 8).

### 3.3. Detailed Analysis of Orientation Estimation

#### 3.3.1. Orientation Estimation During a Short Period (1 s~2 s)

**Figure 9:**
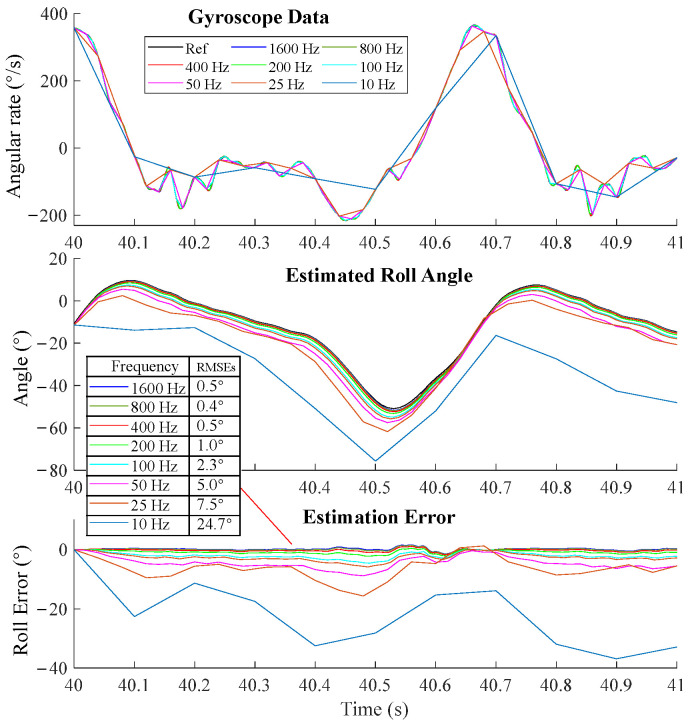
Orientation estimations using the angular velocity integration method with angular velocities at different sampling frequencies. Data were collected from one trial of the walking experiments.

**Figure 10:**
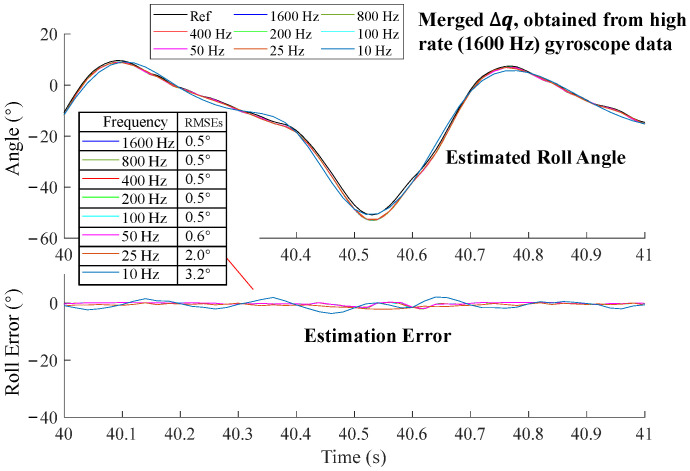
Orientation estimations using the ∆q multiply method with merged ∆q by multiplying several high-rate ${∆q}_{1600Hz}$.

We evaluated the error of the INT algorithm over a short period (1 s~2 s) alongside the whole motion period. For simplicity, we demonstrated roll angle estimation as an example. The initial roll angle was aligned with the reference angle at 40 s. According to orientation estimations using the angular velocity integration method with angular velocities at different sampling frequencies (Figure 9), we can see that the peak error of the 50 Hz roll angle reached nearly 6° in 0.5 s, and errors of 25 Hz and 10 Hz were even bigger. We also calculated the RMSEs of the estimated roll angle of different sampling frequencies. The RMSEs tended to increase as the frequency decreased from 400 Hz to 10 Hz. When merging high rate ∆q into low rate ∆q and then using it for integration, RMSEs of all frequencies exhibited comparable accuracy to 1600 Hz (Figure 10).

#### 3.3.2. Influence of the Sampling Rate of Accelerometer

**Figure 11:**
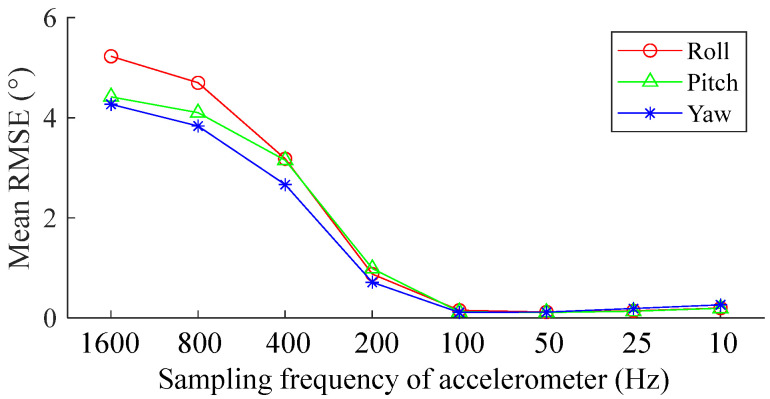
Orientation estimations of the VQF with different sampling frequencies of the accelerometer, with the gyroscope rate fixed at 1600 Hz and the accelerometer data rate ranging from 1600 Hz to 10 Hz. The data were collected from all the walking and running trials.

We investigated the influence of the sampling rate of the accelerometer, selecting the VQF algorithm as the representative algorithm. The trials included both walking and running trials. The gyroscope rate was fixed at 1600 Hz, and the accelerometer data rate ranged from 1600 Hz to 10 Hz. We calculated the mean RMSE across all the trials. The results demonstrated that a higher acceleration rate did not necessarily result in a more accurate orientation estimation. Conversely, errors tended to increase at frequencies exceeding 100 Hz, while accelerometer frequencies ranging from 100 Hz to 10 Hz yielded comparable accuracies (Figure 11).

## 4. Discussion

The purpose of this paper was to investigate how IMU sampling frequency affects orientation estimation and to recommend appropriate low sampling frequencies for human movement analysis, thus reducing power consumption and cost. Experimental tests were performed under different movement conditions. The results demonstrated that a high sampling frequency of the gyroscope can improve the accuracy of strap-down integration and subsequently improve the orientation estimation accuracy, particularly during dynamic movements. The sufficient IMU sampling rates were 100 Hz for walking, 200 Hz for running, and 400 Hz for high-speed cyclic movements (3 Hz).

For the orientation estimation during walking (Figure 3), pitch RMSEs of SEL and VQF tended to increase when sampling frequencies were below 100 Hz. While for the orientation estimation during running, they tended to increase when sampling frequencies were below 200 Hz. For the orientation estimation on the motion testbed, the INT algorithm performed well during slow movements (Figure 6a) when the sampling frequencies were above 50 Hz. However, in the fast movements, the RMSEs increased as the sampling frequency fell below 400 Hz (Figure 6b). The RMSEs of 50 Hz, 25 Hz, and 10 Hz became unacceptable in about six seconds (Figure 7c). Hence, 400 Hz was a sufficient sampling frequency for this state. Interestingly, compared to gyroscope data rates, accelerometer rates appeared less important, with RMSEs even increasing when the accelerometer rate exceeded 100 Hz. Sampling frequencies ranging from 100 Hz to 10 Hz yielded comparable accuracy (Figure 11). A plausible explanation is that when the measured acceleration was distorted by motion acceleration, updating the orientation too frequently using the distorted acceleration introduced more errors because orientation updates based on angular velocity integration were relatively accurate in a short period (Figure 5). These results also underscore the dominance of angular velocity integration in orientation estimation.

Orientation error resulting from lower frequency integration is known as the coning error. It causes angular inaccuracies when the SFA does not integrate the angular velocity fast enough [41,42]. Our results demonstrated that ∆q merged from high rate gyroscope data using the ${INT}_{multiply}$ method effectively compensated for coning error (Figure 8 and Figure 10). To reduce the extra computational requirements of unnecessary high-rate acceleration-based correction, high-rate gyroscope readings can be converted to low-frequency ∆q using the ${INT}_{multiply}$ method, then fed into SFA together with a low-frequency acceleration and magnetic field. This approach can maintain comparable accuracy as a high-frequency sensor fusion. Compared with ${INT}_{add}$, the results of ${INT}_{multiply}$ remain a unit quaternion and do not introduce normalization errors, which is appropriate for merging multiple high-rate gyroscope data. The INT algorithm with high-frequency gyroscope data is also important for yaw estimation because magnetometer-based yaw correction is easily affected by surrounding ferromagnetic materials [31]. In such cases, an accurate INT algorithm provides an effective way to enhance yaw estimation accuracy.

For commonly used wireless wearable IMUs, power consumption is a critical factor in extending the battery life, and smaller data size is important for reliable data transmission. The recommended sufficient sampling frequencies provide an essential reference for developing wearable IMUs or enable a wider choice when selecting off-the-shelf IMUs. Researchers are committed to exploring how low the sampling frequency can be for specific applications [26,43]. Compared with those previous studies, this study provides sufficient sampling frequencies for a broad range of movement speeds and analyzed a wider sampling frequency up to 1600 Hz. In addition, the presented method of merging multiple high-rate gyroscope readings into low-frequency ∆q avoids high-rate raw gyroscope data transmission. Thus, it has beneficial effects on reducing power consumption and increasing the reliability of data transmission.

Validating SFAs in fast-movement conditions is crucial to assess their limitations. Guo et al. [37] proposed a fast Kalman filter algorithm and evaluated its performance in the duration of a 60-s motion trial. The motion was relatively slow and contained some rest states, which may not fully represent its performance in fast-movement scenarios. Caruso et al. [23] compared the performance of ten popular SFAs under optimal tuning parameters. They validated SFAs under slow and fast movements and found that one SFA with optimal parameters did not fit all testing conditions. Nazarahari et al. [19] performed an extensive experimental comparison survey with 36 SFAs. Potter et al. [44] investigated the effects of IMU sampling frequency on gait parameter estimation. However, limited by the hardware or their methods, they did not evaluate whether increasing sampling frequency could improve the accuracy of orientation estimation. Our study analyzed the popular SFAs from the perspective of sampling frequency, providing a comprehensive understanding of how sampling frequency affects orientation estimation accuracy. In our study, the VQF achieved relatively good performance under walking and running conditions, even for the foot IMU (Figure 4). However, under fast movement conditions (Figure 5b), the VQF’s estimations tended to diverge, indicating that movements at 3 Hz were even more challenging than running conditions. Interestingly, although all four SFAs’ estimations became divergent, the INT algorithm with correct initial orientation still performed well, suggesting its effectiveness in challenging conditions. Therefore, we can conclude that these SFAs still require more intelligent adaptive strategies to rely more on gyroscope integration during such highly dynamic movements.

This study still has limitations. First, we only validated the performance of the selected algorithms in walking, running, and cyclic movements with limited movement frequency ranges. Although these validation scenarios included continuous acceleration disturbance, they might not fully represent real-world applications. Further validation experiments should include more applications, such as daily living activities, sports, etc. Second, the IMU used in our experiments was an IMU with dedicated factory calibration. Results may be different when using cheaper wearable IMUs. Future studies should validate the results using a customized, cheaper, high-frequency IMU. Additionally, significant errors in orientation estimation were observed in fast movements using the selected SFAs. This phenomenon highlights the necessity for a new SFA with more intelligent strategies to fully utilize the potential of high-rate angular velocity integration and perform well under these challenging conditions.

## 5. Conclusions

This study investigated the influence of sampling frequency and recommended sufficient sampling frequency across various movement speeds. The experimental results demonstrated that different movements require different preferable sampling frequencies, with IMU sampling frequencies of 400 Hz, 200 Hz, and 100 Hz identified as appropriate for 3 Hz fast movements, running, and walking conditions. The quaternion multiply based integration method can be used to merge high-frequency angular velocity into lower-frequency quaternion increments, reducing computational load while maintaining accuracy. These results could serve as a foundation for developing accurate IMUs or selecting IMUs with appropriate sampling frequencies, thus obtaining a good compromise between accuracy and power consumption or cost.

## Figures and Tables

**Table 1:** Parameters of the selected sensor fusion algorithms.

Algorithm	Parameters
FSM [11] Fan et al.	$\mathrm{Fixed}\;\mathrm{gain}:\;K_{a}=0.2$$,\;K_{m}=0.005$.
ECF [18] Madgwick et al.	$K_{p}=0.5.$
VQF [20] Laidig and Seel	Parameters are the same as the default values in the open-source code.
SEL [36] Seel and Ruppin	tauAcc = 1, tauMag = 3, zeta = 0, accRating = 1.
INT [39]	The initial Euler angles were calculated using accelerometer and magnetometer data during the static period.

## Data Availability

The data presented in this study are available on request from the corresponding author.

## References

[1] Picerno P., Iosa M., D’souza C., Benedetti M.G., Paolucci S., Morone G. (2021). Wearable inertial sensors for human movement analysis: A five-year update. Expert Rev. Med. Devices.

[2] Majumder S., Deen M.J. (2021). Wearable IMU-Based System for Real-Time Monitoring of Lower-Limb Joints. IEEE Sens. J..

[3] Kong W., Sessa S., Cosentino S., Zecca M., Saito K., Wang C., Imtiaz U., Lin Z., Bartolomeo L., Ishii H. Development of a real-time IMU-based motion capture system for gait rehabilitation. Proceedings of the 2013 IEEE International Conference on Robotics and Biomimetics (Robio).

[4] Dahl K.D., Dunford K.M., Wilson S.A., Turnbull T.L., Tashman S. (2020). Wearable sensor validation of sports-related movements for the lower extremity and trunk. Med. Eng. Phys..

[5] Dadashi F., Mariani B., Rochat S., Büla C.J., Santos-Eggimann B., Aminian K. (2014). Gait and Foot Clearance Parameters Obtained Using Shoe-Worn Inertial Sensors in a Large-Population Sample of Older Adults. Sensors.

[6] Dowling A.V., Favre J., Andriacchi T.P. (2012). Inertial Sensor-Based Feedback Can Reduce Key Risk Metrics for Anterior Cruciate Ligament Injury During Jump Landings. Am. J. Sports Med..

[7] Pratt K.A., Sigward S.M. (2018). Detection of Knee Power Deficits Following Anterior Cruciate Ligament Reconstruction Using Wearable Sensors. J. Orthop. Sports Phys. Ther..

[8] Yin Z.-X., Xu H.-M. A wearable rehabilitation game controller using IMU sensor. Proceedings of the 4th IEEE International Conference on Applied System Innovation 2018 (IEEE ICASI 2018).

[9] Nazarahari M., Noamani A., Ahmadian N., Rouhani H. (2019). Sensor-to-body calibration procedure for clinical motion analysis of lower limb using magnetic and inertial measurement units. J. Biomech..

[10] McGrath T., Stirling L. (2022). Body-Worn IMU-Based Human Hip and Knee Kinematics Estimation during Treadmill Walking. Sensors.

[11] Fan B., Li Q., Liu T. (2018). Improving the accuracy of wearable sensor orientation using a two-step complementary filter with state machine-based adaptive strategy. Meas. Sci. Technol..

[12] Benoussaad M., Sijobert B., Mombaur K., Coste C.A. (2016). Robust Foot Clearance Estimation Based on the Integration of Foot-Mounted IMU Acceleration Data. Sensors.

[13] Ju H., Lee M.S., Park S.Y., Song J.W., Park C.G. (2016). A pedestrian dead-reckoning system that considers the heel-strike and toe-off phases when using a foot-mounted IMU. Meas. Sci. Technol..

[14] Nazarahari M., Rouhani H. (2021). 40 years of sensor fusion for orientation tracking via magnetic and inertial measurement units: Methods, lessons learned, and future challenges. Inf. Fusion.

[15] Poddar S., Kumar V., Kumar A. (2017). A Comprehensive Overview of Inertial Sensor Calibration Techniques. J. Dyn. Syst. Meas. Control. Trans. ASME.

[16] Sabatini A.M. (2011). Estimating Three-Dimensional Orientation of Human Body Parts by Inertial/Magnetic Sensing. Sensors.

[17] Madgwick S.O.H., Harrison A.J.L., Vaidyanathan R. Estimation of IMU and MARG orientation using a gradient descent algorithm. Proceedings of the 2011 IEEE International Conference on Rehabilitation Robotics.

[18] Madgwick S.O.H., Wilson S., Turk R., Burridge J., Kapatos C., Vaidyanathan R. (2020). An Extended Complementary Filter for Full-Body MARG Orientation Estimation. IEEE-ASME Trans. Mechatron..

[19] Nazarahari M., Rouhani H. (2021). Sensor fusion algorithms for orientation tracking via magnetic and inertial measurement units: An experimental comparison survey. Inf. Fusion.

[20] Laidig D., Seel T. (2023). VQF: Highly accurate IMU orientation estimation with bias estimation and magnetic disturbance rejection. Inf. Fusion.

[21] De Vries W.H.K., Veeger H.E.J., Baten C.T.M., van der Helm F.C.T. (2009). Magnetic distortion in motion labs, implications for validating inertial magnetic sensors. Gait Posture.

[22] Xsens Technologies B.V (2019). Magnetic Field Mapper Documentation.

[23] Caruso M., Sabatini A.M., Laidig D., Seel T., Knaflitz M., Della Croce U., Cereatti A. (2021). Analysis of the Accuracy of Ten Algorithms for Orientation Estimation Using Inertial and Magnetic Sensing under Optimal Conditions: One Size Does Not Fit All. Sensors.

[24] Romijnders R., Warmerdam E., Hansen C., Welzel J., Schmidt G., Maetzler W. (2021). Validation of IMU-based gait event detection during curved walking and turning in older adults and Parkinson’s Disease patients. J. Neuroeng. Rehabil..

[25] Del Rosario M.B., Lovell N.H., Redmond S.J. (2016). Quaternion-Based Complementary Filter for Attitude Determination of a Smartphone. IEEE Sens. J..

[26] Riddick R., Smits E., Faber G., Shearwin C., Hodges P., van den Hoorn W. (2023). Estimation of human spine orientation with inertial measurement units (IMU) at low sampling rate: How low can we go?. J. Biomech..

[27] Williamson J., Qi L., Lu F., Mohrman W., Li K., Dick R., Shang L. Data sensing and analysis: Challenges for wearables. Proceedings of the 20th Asia and South Pacific Design Automation Conference.

[28] Carratù M., Iacono S.D., Hoang M.L., Pietrosanto A. Energy characterization of attitude algorithms. Proceedings of the 2019 IEEE 17th International Conference on Industrial Informatics (INDIN).

[29] Zhu K., Li D., Li J., Shull P.B. (2024). Real-time IMU-Based Kinematics in the Presence of Wireless Data Drop. IEEE J. Biomed. Health Inform..

[30] Liu X., Wang Z., Li J., Cangelosi A., Yang C. (2023). Demonstration Learning and Generalization of Robotic Motor Skills Based on Wearable Motion Tracking Sensors. IEEE Trans. Instrum. Meas..

[31] Fan B., Li Q., Liu T. (2018). How Magnetic Disturbance Influences the Attitude and Heading in Magnetic and Inertial Sensor-Based Orientation Estimation. Sensors.

[32] Ligorio G., Sabatini A.M. (2015). A Novel Kalman Filter for Human Motion Tracking With an Inertial-Based Dynamic Inclinometer. IEEE Trans. Biomed. Eng..

[33] Bailey J.P., Mata T., Mercer J. (2017). Is the Relationship Between Stride Length, Frequency, and Velocity Influenced by Running on a Treadmill or Overground?. Int. J. Exerc. Sci..

[34] Kobayashi T., Koh M.W.P., Hu M., Murata H., Hisano G., Ichimura D., Hobara H. (2022). Effects of step frequency during running on the magnitude and symmetry of ground reaction forces in individuals with a transfemoral amputation. J. Neuroeng. Rehabil..

[35] Laidig D., Weygers I., Bachhuber S., Seel T. VQF: A Milestone in Accuracy and Versatility of 6D and 9D Inertial Orientation Estimation. Proceedings of the 2022 25th International Conference on Information Fusion (Fusion 2022).

[36] Seel T., Ruppin S. (2017). Eliminating the Effect of Magnetic Disturbances on the Inclination Estimates of Inertial Sensors. IFAC Pap..

[37] Guo S., Wu J., Wang Z., Qian J. (2017). Novel MARG-Sensor Orientation Estimation Algorithm Using Fast Kalman Filter. J. Sens..

[38] Han S., Wang J. (2011). A Novel Method to Integrate IMU and Magnetometers in Attitude and Heading Reference Systems. J. Navig..

[39] Lee J.K., Choi M.J. (2018). Effect of Strapdown Integration Order and Sampling Rate on IMU-Based Attitude Estimation Accuracy. Sensors.

[40] Qureshi U., Golnaraghi F. (2017). An Algorithm for the In-Field Calibration of a MEMS IMU. IEEE Sens. J..

[41] Zhang L., Zhang T., Wang M., Wang J., Li Y. (2020). A High-Order Coning Error Compensation Algorithm Under High Rate Maneuvering. IEEE Sens. J..

[42] Wang M., Wu W., Wang J., Pan X. (2015). High-order attitude compensation in coning and rotation coexisting environment. IEEE Trans. Aerosp. Electron. Syst..

[43] Pfau T., Reilly P. (2021). How low can we go? Influence of sample rate on equine pelvic displacement calculated from inertial sensor data. Equine Vet. J..

[44] Potter M.V., Ojeda L.V., Perkins N.C., Cain S.M. (2019). Effect of IMU Design on IMU-Derived Stride Metrics for Running. Sensors.

